# Multifactorial analysis of simultaneous organelle movement reveals cell-specific motility of peroxisomes and mitochondria

**DOI:** 10.1093/plphys/kiag119

**Published:** 2026-02-28

**Authors:** Amanda M Koenig, Katarzyna Krawczyk, Calvin H Huang, Yuh-Ru Julie Lee, Bo Liu, Jianping Hu

**Affiliations:** Michigan State University-Department of Energy Plant Research Laboratory, Michigan State University, East Lansing, MI 48824, United States; Department of Plant Biochemistry, Heinrich Heine University, Düsseldorf 40225, Germany; Department of Plant Biology, College of Biological Sciences, University of California, Davis, CA 95616, United States; Department of Plant Biology, College of Biological Sciences, University of California, Davis, CA 95616, United States; Department of Plant Biology, College of Biological Sciences, University of California, Davis, CA 95616, United States; Michigan State University-Department of Energy Plant Research Laboratory, Michigan State University, East Lansing, MI 48824, United States; Department of Plant Biology, Michigan State University, East Lansing, MI 48824, United States

## Abstract

The movement, distribution, and interactions of organelles are cell-type specific, responding to fluctuating metabolic and environmental cues and governing the efficiency of plant physiology and stress response. The directional motility of various plant organelles is predominantly driven by the actomyosin system, yet the distinct functionality of these organelles across plant tissues presupposes organelle-specific regulation of motility, which requires the detection of subtle shifts in dynamics. Meanwhile, studies that comprehensively characterize and directly compare the simultaneous movement of multiple types of organelles within the same cell are limited. Here, we visualized peroxisomes, mitochondria, chloroplasts, Golgi bodies, and actin filaments simultaneously in tobacco (*Nicotiana tabacum*) to evaluate organelle organization and motility within the context of one another. Quantitative analysis of multiple motility factors enabled us to identify peroxisome motility in tobacco mesophyll as distinct from other organelles. Further analysis in Arabidopsis (*Arabidopsis thaliana*) revealed that both mitochondria and peroxisomes are slower in mesophyll cells compared to epidermis in normal growth conditions, but their motility patterns are unique from one another across leaf tissue after plants experienced conditions that induce photorespiration, a metabolic pathway requiring the concerted action of chloroplasts, peroxisomes, and mitochondria. Our quantitative analysis of thousands of organelles across species, cell type, and physiological conditions unveils distinct modulation of motility according to organelle identity and function. The extensive combinatorial characterizations of plant organelle movement provide a fundamental resource for the future discovery of molecular mechanisms driving the movement and distribution of diverse organelles.

## Introduction

Organelle motility and positioning are paramount to the proper organization and efficient function of cells. Various cell types require distinct space allocation and arrangements of organelles according to their roles in the plant (Kang et al. 2022; Koenig et al. 2023). For example, chloroplasts occupy more space in the mesophyll, the tissue responsible for photosynthesis, compared to other cell types (Larkin 2024). Moreover, the specific role mesophyll cells play in photosynthesis can influence organelle positionality. In C3 plants like rice, mitochondria are arranged more internally between the peripheral chloroplasts and the vacuole, likely to facilitate the recapture of photorespiratory CO_2_ into the Calvin–Benson cycle (CBC). By contrast, in many C4 grasses, in which the CBC occurs in bundle sheath cells, mitochondria are frequently observed between chloroplasts and the cell wall (Hatakeyama and Ueno 2017). Further, organelle distribution is dynamic and shifts in response to environmental conditions, often in a cell-type-dependent manner (Koenig et al. 2023). One example is photorelocation, which is uniquely regulated between epidermal and mesophyll cells. In the dark, chloroplasts in the epidermal pavement cells and mesophyll inversely redistribute toward the top and bottom periclinal walls, respectively, and the nucleus position is phototropin-dependent in the mesophyll and phototropin-independent in the epidermis (Islam et al. 2009; Iwabuchi et al. 2010; Wada 2016).

Plant organelle motility and distribution are governed largely by actomyosin. The Myosin XI motors facilitate organelle transport along actin filaments, whose density and structure impact directional movement at least for peroxisomes (Perico and Sparkes 2018; Huang et al. 2026). Plants deficient in functionally redundant Myosin XI proteins exhibit impaired growth and immunity, linking actomyosin-dependent organelle movement to plant health (Peremyslov et al. 2010; Yang et al. 2014). Light, temperature, CO_2_, metals, hormones, and reactive oxygen species (ROS) are known stimuli for organelle movement (Koenig et al. 2023). Molecular regulators of organelle-specific movement in plants, such as Chloroplast Unusual Positioning 1 (CHUP1) for chloroplasts (Kong et al. 2024), Myosin Recruitment Factor 7 (MRF7) for Golgi (Perico et al. 2021), WPP motif-interacting proteins (WITs) for nuclei (Tamura et al. 2013), and the Miro2 GTPase for ER-mediated mitochondrial motility (White et al. 2020) have been identified. Organelle motility varies across and within cell types according to metabolic demands. For example, pollen tube elongation requires a unique distribution of mitochondria to satisfy the energy demand of tip growth (Cai 2022; Boussardon et al. 2025). Organelle-specific movement drives unique spatial rearrangements within and across cell types, yet studies of cell-specific organelle motility are limited. Further, characterization of organelle motility across organelle identities in the context of each other can provide a direct comparison for understanding their independent regulation, yet published reports have visualized the dynamics of 1 or 2 types of organelles instead of multiple organelles detected simultaneously in living cells (Avisar et al. 2008; Jaipargas et al. 2016).

Photorespiration is a metabolic process occurring by the concerted action of chloroplasts, peroxisomes, mitochondria, and the cytosol (Eisenhut et al. 2019). Through this pathway, 2-phosphoglycolate (2-PG)—the oxygenation product of the photosynthetic enzyme ribulose 1,5-bisphosphate carboxylase-oxygenase—is salvaged to 3-phosphoglycerate (3-PGA) and recycled back to the CBC (Eisenhut et al. 2019). The arrangement and proportions of photorespiratory organelles vary between bundle sheath and mesophyll cells across species (Yoshimura et al. 2004). Besides triggering alternative photorespiratory routes (Jiang et al. 2025), photorespiratory conditions, like high light, are correlated with the aggregation of chloroplasts, peroxisomes, and mitochondria, likely to expedite metabolite exchange among these organelles (Jaipargas et al. 2016). However, how these photorespiratory organelles move in different cell types and whether their movement patterns are associated with the physiological functions of the various cells have not been explored.

To compare the movement patterns for 2 key photorespiratory organelles—peroxisomes and mitochondria—in different cell types, here we observed their motility alongside a nonphotorespiratory organelle, the Golgi apparatus (Kang et al. 2022). Visualizing these organelles within the same cell simultaneously allowed us to directly compare their quantity and motility across the leaf epidermis and mesophyll. Multifactorial analysis showed that peroxisomes move distinctly from other organelles in tobacco mesophyll. Further analysis of Arabidopsis (*Arabidopsis thaliana*) transgenic plants revealed that peroxisomes and mitochondria move more slowly in the chloroplast-dense mesophyll than in epidermis under normal growth conditions, and their motility patterns are unique from one another across leaf tissue after plants were treated with photorespiration-inducing conditions, yet such a distinct motility pattern is not shown in root epidermis. Our findings indicate that not only cell topography but also, critically, organelle function and physiology influence organelle-specific mobility, thus establishing a foundational characterization of organelle movement that enables the evaluation of subtle changes in organelle-specific motility for the elucidation of their molecular mechanisms.

## Results

### The distribution and motility of peroxisomes are distinct from those of mitochondria and the Golgi in tobacco

**Figure 1:**
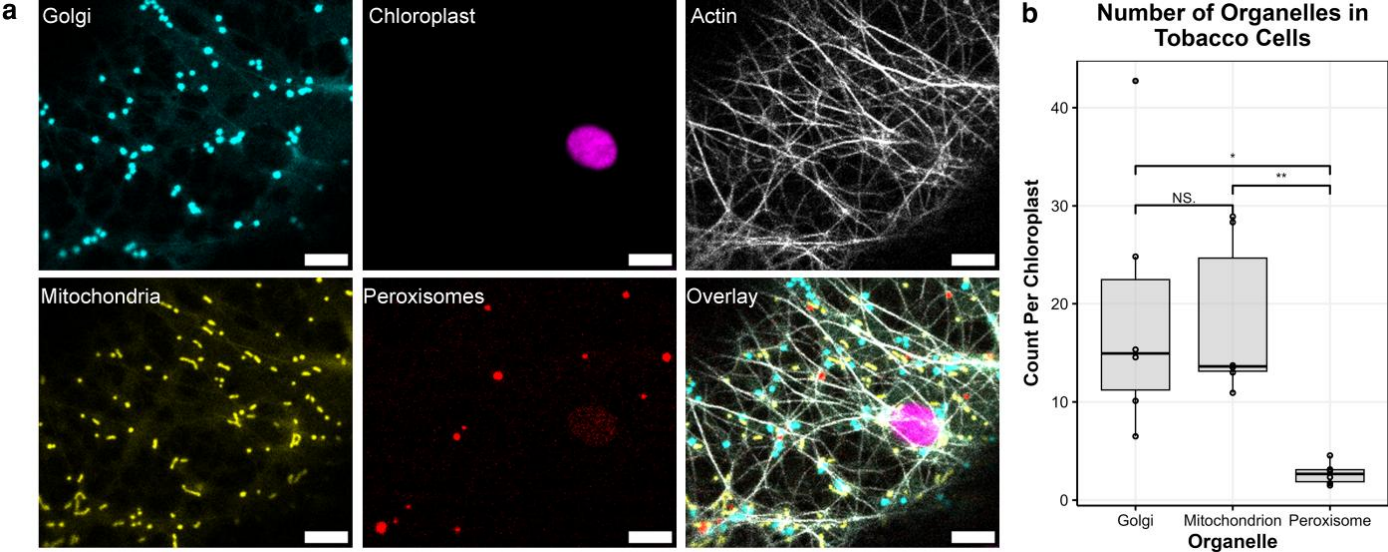
Simultaneous live imaging of multiple fluorescent organelle markers reveals the subcellular landscape of tobacco cells. **a)** Fluorescent markers for organelles and F-actin in a representative tobacco leaf epidermal cell. **b)** Quantification of peroxisomes, mitochondria, and Golgi across both epidermis and mesophyll. Total numbers were normalized to the number of chloroplasts shown as a boxplot (center line, median; box limits, upper and lower quartiles; whiskers, 1.5× interquartile range; points, individual datapoints). The sizes, morphologies, and distribution of Golgi (GmMan1^49aa^-eCFP, cyan), chloroplasts (chlorophyll autofluorescence, magenta), actin (Lifeact-eGFP, white), mitochondria (ScCOXIV^29aa^-eYFP, yellow), and peroxisomes (mScarlet-I-SRL, red) were visualized within the same cell for direct qualitative comparison. Significance was determined by Welch's *t*-test: *P < 0.05, **P < 0.01.

To compare the motility of photorespiratory organelles in the context of nonphotorespiratory organelles, we simultaneously visualized Golgi bodies, mitochondria, and peroxisomes—organelles often used to assess actomyosin-mediated movement (Peremyslov et al. 2008, 2010; Prokhnevsky et al. 2008)—along with chloroplasts and actin filaments, in tobacco (*Nicotiana tabacum*) (Fig. 1a) transiently expressing previously reported markers (Nelson et al. 2007; Koenig et al. 2023). Similar to previous observations, Golgi and peroxisomes appear mostly spherical, whereas mitochondria are both circular and rod-shaped, consistent with previously reported mitochondrial morphologies (Fig. 1a) (Pan et al. 2014a, 2014b). For organelle quantifications, we normalized the number of organelles to the total chloroplasts in each image to directly compare organelle counts quantified using the ImageJ Cell Counter plugin. Mature tobacco leaves contained comparable quantities of mitochondria and Golgi, and strikingly, far fewer peroxisomes (Fig. 1b, Table S1), all within the reported ranges of these organelles in plants (Kang et al. 2022). These proportions are maintained across leaf epidermis and mesophyll (Fig. S1).

**Figure 2:**
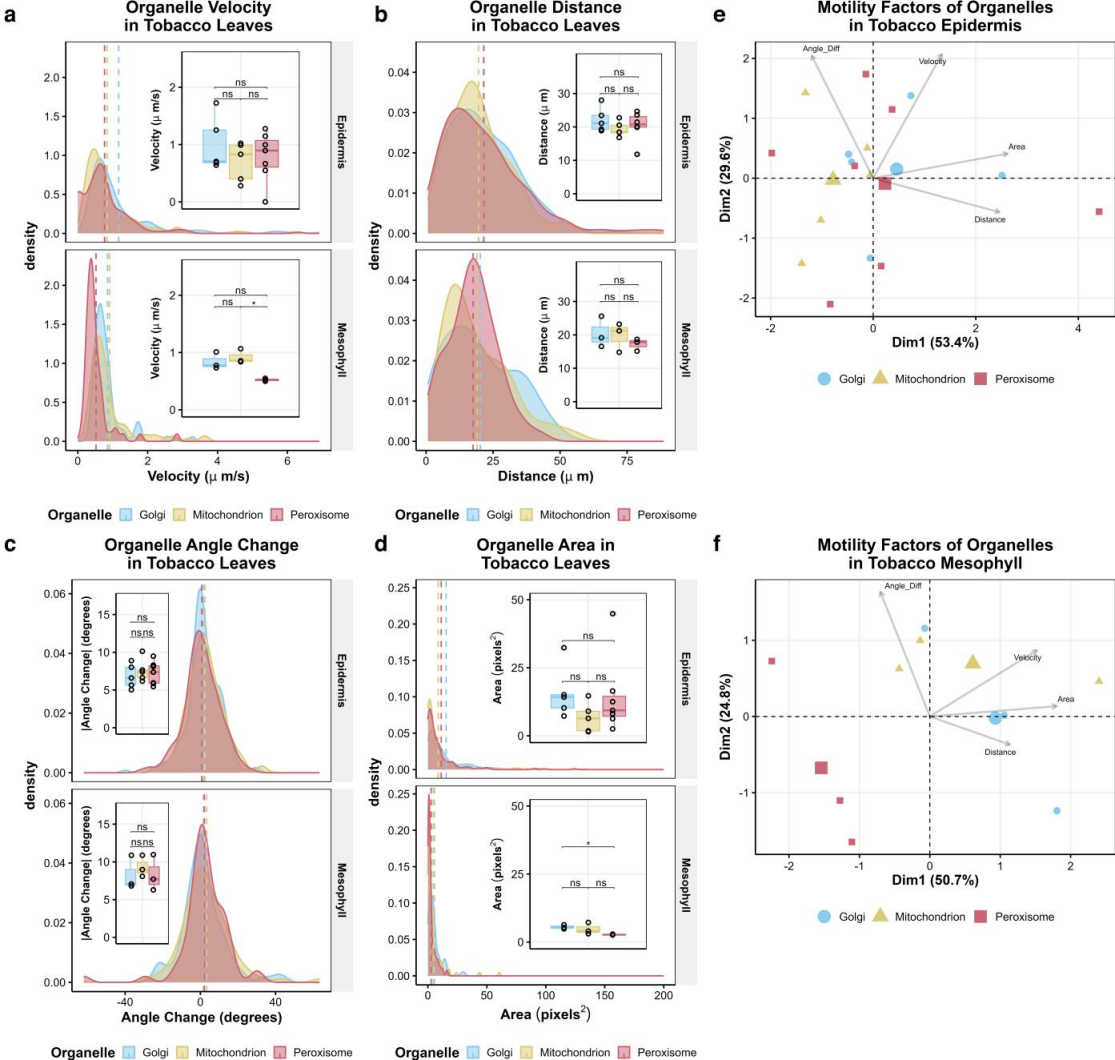
Peroxisome motility is distinct from that of mitochondria and Golgi bodies in tobacco mesophyll. **a** to **d)** Organelle motility represented by velocity, distance, angle change, and area, revealing reduced mobility for peroxisomes compared with mitochondria and the Golgi. The motility factor for each organelle track is shown as a density plot with the average for each organelle designated by a vertical dotted line. The average motility across each plant sample is plotted as an inset boxplot (center line, median; box limits, upper and lower quartiles; whiskers, 1.5× interquartile range; colored points, outliers; black points, individual datapoints). Significance for density plots was determined by ANOVA and Welch's *t*-test, and values are reported in the supplementary data. Significance for inset boxplots was determined by Welch's *t*-test with Bonferroni correction: *P < 0.05, **P < 0.005, ***P < 0.0005. **e, f**) Principal component analysis (PCA) of the average velocity, angle change, area, and distance. While the 3 organelles are indistinguishable by motility pattern in the epidermis (e), peroxisomes cluster distinctly from mitochondria and Golgi in the mesophyll (f). For each organelle, 3 to 7 biological replicates were analyzed. Each point represents organelle data from an individual plant, and the centroid is represented by the large icon for each organelle. The eigenvectors (gray) indicate the correlation of variables, where increased perpendicularity indicates less correlation, similarly oriented arrows are positively correlated, and oppositely oriented arrows indicate negatively correlated variables.

For a comprehensive comparison of the movement patterns, we imaged these organelles simultaneously in both epidermis and mesophyll every 250 ms for 1 min, tracked their movement with MTrackJ (Videos S1 to S8), and analyzed multiple motility factors, including velocity, distance, angle change, and area traveled (Fig. 2, Tables S2, S3). Motility patterns are highly heterogeneous, especially in epidermis, as evidenced by the range of average velocities—Golgi bodies from 0.22 to 6.9 µm/s, mitochondria from 0.13 to 4.7 µm/s, and peroxisomes from 0 to 6.3 µm/s—and distances (Fig. 2a, b, Table S3). Because this complex heterogeneity can be obscured by overall averages of independent plant samples (box plots), we compared movement parameters of each individual organelle (density plots) to reveal intricate differences in motility. According to individual motility factors, Golgi bodies have a higher average velocity (1.17 µm/s) and a greater proportion of them move linearly with an angle change near 0° compared to both peroxisomes (0.77 µm/s) and mitochondria (0.84 µm/s) in the epidermis (Fig. 2a, c, Table S2). Peroxisomes were observed at comparable, or faster, maximum velocities compared with Golgi and mitochondria in the epidermis. However, peroxisome motility is distinct from both mitochondria and Golgi in the mesophyll, as peroxisomes have a minimum velocity of 0.099 µm/s, which is less than half that of Golgi (0.33 µm/s) and mitochondria (0.21 µm/s), and are more frequently observed at lower velocities compared to mitochondria and Golgi, as well as epidermal peroxisomes (Fig. 2a). Further, peroxisomes are significantly slower, at 0.52 µm/s on average, than mitochondria (at 0.91 µm/s) and travel across half the cell area as Golgi bodies in the mesophyll (Fig. 2a, d). Although principal component analysis of velocity, distance, angle change, and area does not resolve organelles in the epidermis into discrete groups (Fig. 2e), peroxisomes are clustered separately from mitochondria and Golgi in the mesophyll (Fig. 2f), suggesting peroxisome movement is unique from the other 2 organelles, particularly in mesophyll cells.

**Figure 3:**
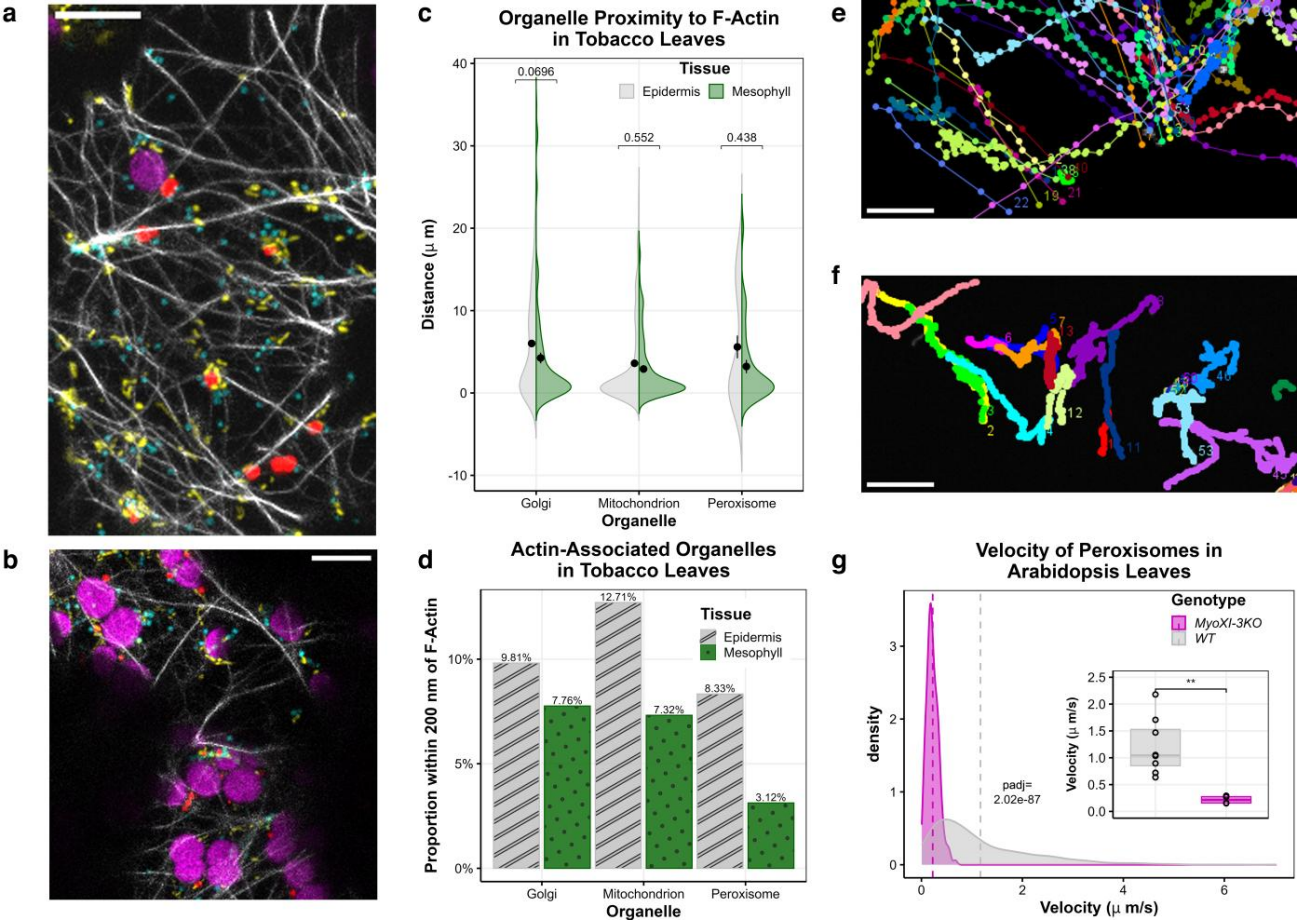
Organelle dependence on actomyosin varies across leaf cell types. **a, b**) Maximum Z-projections of Golgi (GmMan1^49aa^-eCFP, cyan), chloroplasts (chlorophyll autofluorescence, magenta), actin (Lifeact-eGFP, white), mitochondria (ScCOXIV^29aa^-eYFP, yellow), and peroxisomes (mScarlet-I-SRL, red), visualized in the same cells of tobacco. Z-series of the cortex of epidermis (a) and mesophyll cells (b) were analyzed with ImageJ DiAna. **c, d**) Quantification of organelle proximity to F-actin in tobacco. The closest distance from the center of each organelle to the edge of the nearest F-actin filament is reported (c) with the distance for each organelle represented by the violin plots (*n* > 300 for Golgi and mitochondria, *n* > 50 for peroxisomes). The black dot and error bars show the mean distance of the organelles ± standard error. Significance was determined by a Bonferroni-corrected Welch's *t*-test, and adjusted *P*-values are reported on the graph. Note that the appearance of negative proximity measurements is a consequence of the untrimmed violin plot. The proportion of each organelle within a cell type that is within 200 nm of an F-actin filament is also shown (d). **e** to **g**) Visualization and analysis of myosin-dependent peroxisome motility in Arabidopsis. Peroxisomes in leaf epidermis of wild type (WT) (e) and myosin triple knockout (*MyoXI-3KO*) (f) lines were visualized and tracked. The individual velocity of each organelle track is shown as a density plot with the average for each organelle designated by a vertical dotted line, and the average velocity across each plant sample is plotted as an inset boxplot (center line, median; box limits, upper and lower quartiles; whiskers, 1.5× interquartile range; colored points, outliers; black points, individual datapoints) (g). Significance for density plots was determined by a Bonferroni-corrected Welch's *t*-test, and adjusted *P*-values are reported on the graph. Significance for inset boxplots was determined by Student's *t*-test * *P* < 0.05, **P < 0.01. Data represent ≥4 biological replicates, 660 and 170 peroxisomes in wild type and *MyoXI-3KO*, respectively. Scale bars represent 10 µm.

To evaluate each organelle's utilization of actomyosin across cell types, we also compared the proximity of Golgi, peroxisomes, and mitochondria to F-actin in the epidermis or mesophyll cell cortexes (Fig. 3). Using ImageJ plugin DiAna (Gilles et al. 2017), we first measured the distances from the center of each organelle to the edge of the nearest actin filament; a total of 330 Golgi, 304 mitochondria, and 56 peroxisomes were analyzed. The average distance between the organelles and actin is ∼3 to 6 µm, which is not significantly different between epidermis and mesophyll (Fig. 3c). Furthermore, we calculated the proportion of each type of organelle that is within 200 nm of F-actin: ie the approximate length from the motor domain to the cargo-carrying domain of Myosin XI proteins (∼50 nm) (Tominaga and Nakano 2012) plus the length of any possible adaptor proteins to the center of the organelle itself. More epidermal organelles are closely associated with actin filaments compared to their counterparts in the mesophyll, a pattern that was especially pronounced for peroxisomes (8.33% in the epidermis vs. 3.12% in the mesophyll, Fig. 3d). These data indicate that fewer organelles may be associated with Myosin XI motors in the mesophyll than in the epidermis.

### Peroxisomes and mitochondria are less mobile in Arabidopsis mesophyll

Since peroxisomes exhibited disparate quantity, distribution, and motility from mitochondria and Golgi bodies in tobacco mesophyll, we further assessed leaf peroxisomes in Arabidopsis in which myosin mutants are available. Although an F-actin marker was used in tobacco, we assessed organelle mobility irrespective of actin association. Previous studies showed significantly reduced velocity of peroxisome movement in the Myosin XI triple (*Myosin XI-1, Myosin XI-2*, and *Myosin XI-K*; *MyoXI-3KO* in this study) knockouts (Peremyslov et al. 2010). To determine how peroxisomes behave in myosin mutants with our multifactorial motility analysis, we introduced a peroxisome marker (Koenig et al. 2023; Huang et al. 2026) into *MyoXI-3KO* and wild-type backgrounds (Fig. S2). Leaf epidermis of 2-week-old T3 seedlings was imaged every 500 ms for 5 min, with all peroxisomes (∼40 to 160 peroxisomes/video) tracked by MTrackJ (Fig. 3e, f, Video S9). Consistent with previous reports, peroxisome velocity and displacement, the latter of which is quantified by the maximum distance between 2 consecutive points, were greatly reduced in leaf epidermis in *MyoXI-3KO*, with averages of 0.22 µm/s and 6.87 µm, respectively, compared to the WT at 1.17 µm/s and 15.17 µm (Fig. 3g, Figure S2a, Table S4) (Peremyslov et al. 2010). However, the cumulative distance, which is a sum of the distances between each consecutive time point and, thus, accounts for both directional and oscillatory movements, is not significantly different between WT (23.48 µm) and *MyoXI-3KO* (21.88 µm) peroxisomes (Fig. S2b), indicating that this motility factor may be impacted by additional regulators. Therefore, to account for any modulators of organelle motility and present a comprehensive assessment of movement patterns, we kept characterizing organelle mobility in Arabidopsis irrespective of actin association.

We then assessed peroxisome motility between Arabidopsis epidermis and mesophyll. Peroxisomes in the 2 independent WT marker lines exhibit a broader range of motility factor values in the epidermis, whereas peroxisome movements in the mesophyll are more frequently observed at lower values for velocity, distance, and area (Fig. S3), the average of which is significantly lower compared to the epidermis (Table S4). We reasoned that the variation in peroxisome motility patterns between epidermis and mesophyll may be due to both actomyosin-dependent and actomyosin-independent mechanisms.

**Figure 4:**
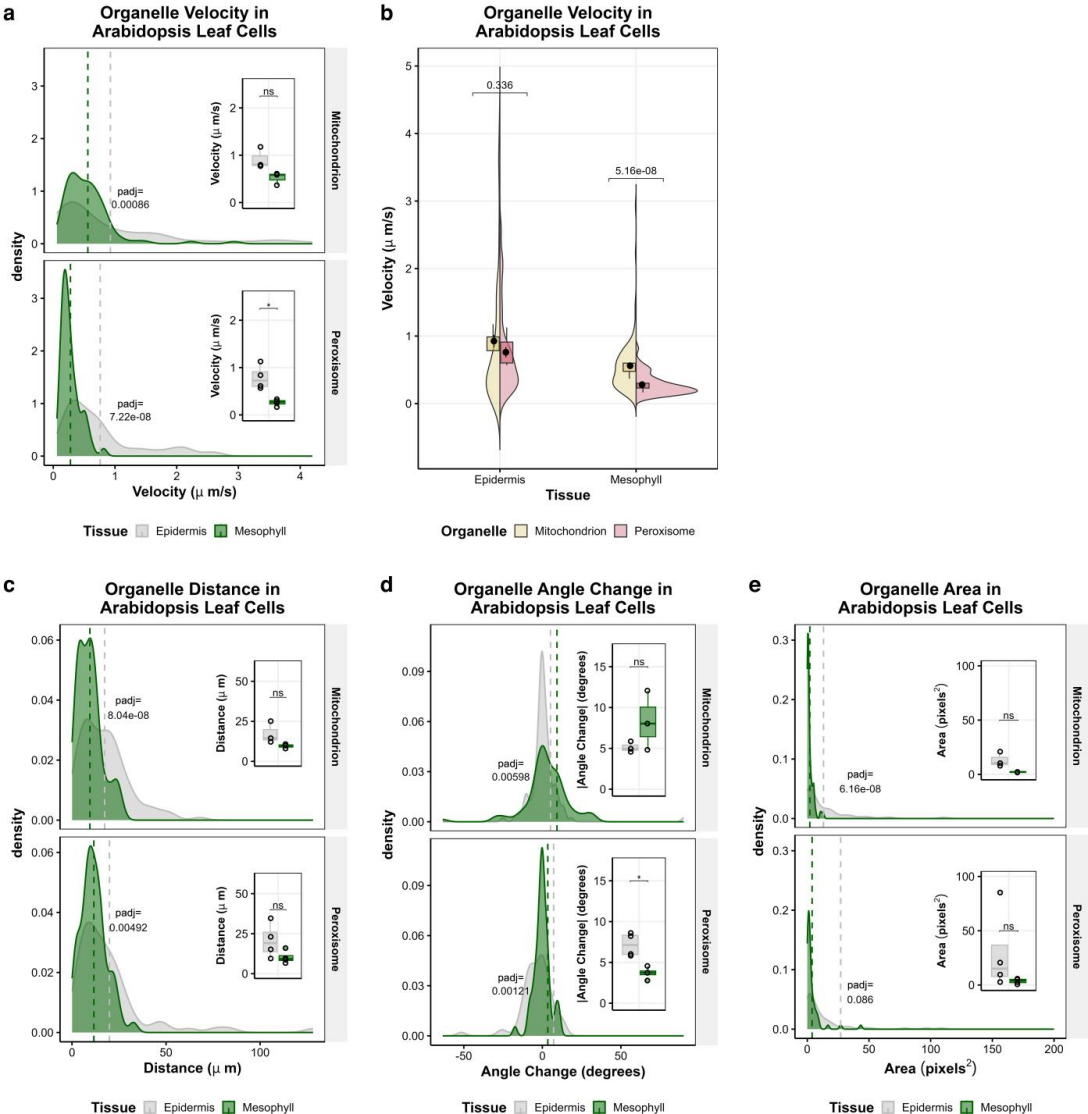
Both mitochondria and peroxisomes are less mobile in the mesophyll than epidermis in Arabidopsis leaves under normal growth conditions. Comparison of the velocity (**a, b**), distance (c), angle change (d), and area (e) of peroxisomes and mitochondria across Arabidopsis leaf epidermis and mesophyll. The individual motility factors for each organelle track are shown as a density (**a, c** to **e)** or split violin (b) plot, with the average for each organelle designated by a vertical dotted line or a black dot, respectively. The average motility across each plant sample is plotted as a boxplot (center line, median; box limits, upper and lower quartiles; whiskers, 1.5× interquartile range; colored points, outliers; black points, individual datapoints). Significance for density and split violin plots was determined by a Bonferroni-corrected Welch's *t*-test, and adjusted *P*-values are reported on the graph. Significance for inset boxplots was determined by Welch's *t*-test with Bonferroni correction: * *P* < 0.05, **P < 0.005, ***P < 0.0005.

To test whether this reduction in peroxisome motility is organelle-specific, we compared peroxisomes and mitochondria in Arabidopsis leaves. Two-week-old plants from mitochondrion and peroxisome CFP marker lines (Nelson et al. 2007) were visualized every 250 ms in the epidermis and in the mesophyll of the same leaf sample (Videos S10 to S11), followed by organelle tracking and motility quantification. Consistent with observations in tobacco (Fig. 1), Arabidopsis peroxisomes, with an average velocity of 0.28 µm/s, are slower than mitochondria (at 0.56 µm/s) in the mesophyll, in which both organelles exhibit lower velocity, shorter distances, and reduced area compared to their counterparts in the epidermis (Fig. 4, Table S5). Interestingly, more mitochondria move linearly, as indicated by an angle change near 0°, in the epidermis, while more peroxisomes exhibit linear movement in the mesophyll (Fig. 4d).

Overall, both peroxisomes and mitochondria are less mobile in mesophyll cells compared to the epidermis, although linear movement patterns are more frequently observed in the epidermis for mitochondria and in the mesophyll for peroxisomes, indicating distinct regulation between organelles and cell types.

### Photorespiration-induced shifts in mitochondrial and peroxisomal motility differ across cell types

Since both mitochondria and peroxisomes move more slowly in the mesophyll, we wanted to understand which physiological process may drive this reduction in motility. As the major location for photosynthesis, mesophyll cells are dominated by chloroplasts. While the density of chloroplasts in the mesophyll cells may result in a crowded environment, the epidermis is likely similarly crowded by other organelles, mostly the vacuole (Erickson et al. 2024). Further, chloroplasts have been shown to influence the positioning and movement of other organelles (Oikawa et al. 2021). Finally, contact among mitochondria, peroxisomes, and chloroplasts increases during photorespiratory conditions such as high light (Oikawa et al. 2015). Therefore, we assessed mitochondrial and peroxisomal movement immediately after plants experienced photorespiration-inducing conditions.

After growing in normal growth conditions (∼100 µmol/m^2^s) for 7 d, seedlings were moved into high light (∼700 µmol/m^2^s) to grow for another week. Two days before imaging, the plates were wrapped by three layers of parafilm to restrict gas exchange and thereby reduce CO_2_ access to rubisco (Vanderauwera et al. 2012; Kerchev et al. 2015; Waszczak et al. 2016). Organelles in the epidermis and mesophyll of the same leaf and in the epidermal cells of the root maturation zone were imaged every 250 ms, followed by movement tracking and quantification. Plants grown continuously in normal growth conditions were used as the control.

**Figure 5:**
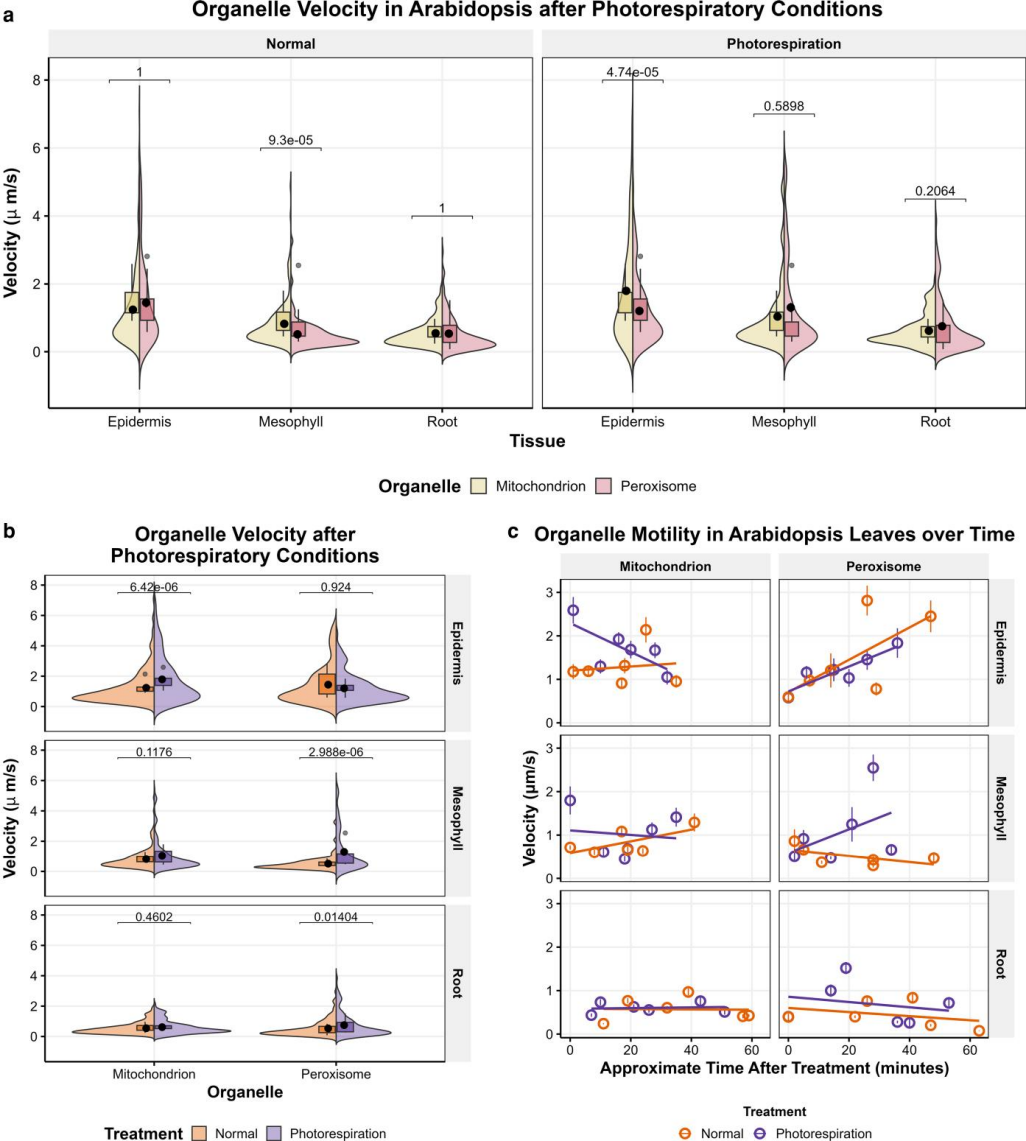
The motility of peroxisomes and mitochondria coordinates in the mesophyll in response to photorespiration-inducing conditions. **a, b**) Violin plots of the velocity of mitochondria and peroxisomes in leaf epidermal and mesophyll and root epidermal cells following photorespiration-inducing conditions. **c**) Average velocity of peroxisomes and mitochondria for each plant sample at different times points after plants were treated with photorespiration-inducing conditions. The velocity for each organelle track is shown as a violin plot with the average for each organelle designated by a black dot with bars indicating the standard error, and the average velocity across each plant sample (*n* = 6) is shown as a boxplot (center line, median; box limits, upper and lower quartiles; whiskers, 1.5× interquartile range; colored points, outliers; black points, individual datapoints) (a, b). Significance was determined by a Bonferroni-corrected Welch's *t*-test, and adjusted *P*-values are reported on the graph. The data represent >700 peroxisomes and >1,300 mitochondria.

Under normal conditions, peroxisomal and mitochondrial velocities are not statistically different in leaf and root epidermis, and peroxisomes are significantly slower than mitochondria in the mesophyll (Fig. 5a, Table S6), consistent with results from our earlier experiments (Figs 2a and 4a, b). Interestingly, following photorespiratory conditions, mitochondria, with an average velocity of 1.79 µm/s, move faster than peroxisomes, at 1.20 µm/s, in the leaf epidermis, whereas mitochondria and peroxisomes exhibit similar motility—1.03 µm/s and 1.30 µm/s, respectively—in leaf mesophyll (Fig. 5a). The motility of mitochondria and peroxisomes relative to one another in the roots remains consistent following treatment with photorespiratory conditions (Fig. 5a). Taken together, these data suggest that mitochondrial and peroxisomal motility is responsive to photorespiration-inducing conditions in photosynthetic leaf tissue but not in roots. Further, following high-light and limited gas exchange, the overall velocity of mitochondria and peroxisomes may coordinate in mesophyll cells, where chloroplasts, mitochondria, and peroxisomes communicate and interact to facilitate photorespiration.

To observe the changes in motility throughout the recovery period, we analyzed the velocities of organelles at different time points during plant recovery from the photorespiration-inducing conditions. Interestingly, the significant increase in mitochondrial motility in the leaf epidermis following photorespiration abates during recovery, whereas peroxisome velocity is similar to its baseline slower speed immediately after removal from high-light/limited gas exchange and then trends upward over time after photorespiratory stress treatment (Fig. 5b, Fig. S4). The distinct patterns of mitochondria and peroxisome velocity suggest organelle-specific regulation of their motility in response to the changing environment.

## Discussion

In this work, we characterized plant organelle motility extensively and collectively as a foundation to uncover nuanced modulation of organellar dynamics, using a large-scale approach to assess organelle movement by multiple motility factors across species, cell types, and physiological conditions. Precise temporal resolution (250 to 500 ms intervals) was used to quantify the movement and distribution patterns of nearly 10,000 organelles—2015 Golgi bodies, 3,452 mitochondria, and 3,889 peroxisomes—in leaf epidermis and mesophyll of tobacco and Arabidopsis as well as Arabidopsis root epidermis. Quantification was conducted in the context of multiple organelles simultaneously and under varying environmental conditions.

Although Golgi, peroxisomes, and mitochondria are often assessed to elucidate actomyosin-dependent organelle motility (Peremyslov et al. 2008, 2010; Prokhnevsky et al. 2008), few studies directly compare motility between these organelles within the same cell. A previous study simultaneously visualized Arabidopsis mitochondria, peroxisomes, chloroplasts, and the endoplasmic reticulum to investigate aggregation and inter-organellar interactions, but reported minimal and only qualitative assessments of organelle movement (Jaipargas et al. 2016). Peroxisomes and Golgi bodies have been previously visualized simultaneously in tobacco epidermis, and consistent with our findings, Golgi bodies were found to move more rapidly than peroxisomes (Avisar et al. 2008). While Golgi movement supports cargo trafficking and export as part of the secretory pathway throughout plant tissue, peroxisomes and mitochondria, as energy organelles, move at least in part in response to metabolic demand, with specialized roles such as photorespiration in photosynthetic tissue (Kang et al. 2022). Therefore, we evaluated physiologically related organelles—mitochondria and peroxisomes—alongside the morphologically similar Golgi with high temporal resolution (250 ms intervals) to directly compare several motility factors both individually and combinatorially. Surprisingly, the number of peroxisomes per chloroplast is only 1/7 that of mitochondria and Golgi in tobacco leaves (Fig. 1b, Table S1), indicating that leaf peroxisomes are more specialized in function and that their distribution and motility must be deliberate in response to cellular demand. Peroxisomes also move distinctly from the other 2 types of organelles specifically in the mesophyll (Figs 1 and 2), suggesting unique regulation of peroxisome motility especially in photosynthetic cells, possibly due to its predominant role as a photorespiratory compartment while other organelles perform more diverse functions in the same mesophyll.

Organelle motility can be the result of cytoplasmic streaming, as in the extremely rapid directional movement often observed in elongated cells like root hairs, trichomes, and pollen tubes (Jedd and Chua 2002). However, the heterogeneity of motility patterns we observe across organelle identity and cell type (Figs 2 to 4) supports direct organelle translocation by motors in an organelle-specific manner. Although the average distance of each organelle to the nearest F-actin is not statistically different between leaf epidermis and mesophyll (Fig. 3c), the proportion of organelles within 200 nm of an actin filament is lower in the mesophyll compared to the epidermis (Fig. 3d). Organelles within this distance of the cytoskeleton could reasonably be associated with motors, which enable faster movement. In this study, we tracked all organelles irrespective of their visual proximity to actin filaments. The increased proportion of immobile Golgi bodies, peroxisomes, and mitochondria in the mesophyll (Figs 2 and 4) may be partially attributed to organelles disassociated from actomyosin.

We further found leaf cell-specific morphology and physiology to correlate with differential motility patterns of mitochondria and peroxisomes, after comparing these 2 organelles in the pavement cells of the epidermis and the spongy mesophyll. The predominant role of the leaf epidermis is as a protective boundary against pathogens and abiotic stresses and as a gateway for transpiration and gas exchange. The pavement cells, in particular, are widely considered as the mechanical supports to provide structure and organization to the leaf epidermis (Zuch et al. 2022). Contrastingly, the mesophyll as the site of photosynthesis contains approximately 10 times more chloroplasts, which are also larger in size than those in pavement cells (Barton et al. 2018). Chloroplasts have a commanding influence on the cellular landscape and dynamics, both in the space they occupy and the physiological impact on organelle movement. For example, mitochondrial motility shifts from actin-dependent directional movement to Brownian-type wiggling patterns when in proximity to chloroplasts (Oikawa et al. 2021). We observed a higher proportion of mitochondria with linear motility in the epidermis, which contain substantially fewer chloroplasts than the mesophyll (Fig. 4d).

Given that photorespiration requires the metabolic exchange among peroxisomes, chloroplasts, and mitochondria, the domineering influence of chloroplasts enriched in the mesophyll likely affects the dynamics of its organellar partners. In fact, we found in this study that peroxisomal and mitochondrial motility patterns shift possibly to coordinate during photorespiration, specifically in mesophyll cells. The average velocities of mesophyll mitochondria (1.03 µm/s) and peroxisomes (1.30 µm/s) following high-light and limited gas exchange conditions are statistically the same (Fig. 5a), likely reflecting their collaborative role in photorespiration. However, peroxisome and mitochondrion motility patterns are not always correlated, even in response to the same stress treatment. For example, following photorespiration, peroxisomes and mitochondria behave distinctly in the leaf epidermis. Mitochondrial speeds are faster, at 1.79 µm/s on average, compared to normal conditions at 1.24 µm/s, which taper back to baseline velocity over time. On the other hand, peroxisome velocity in the leaf epidermis is statistically unaffected by photorespiration-inducing conditions (1.20 µm/s vs. 1.44 µm/s) but rather trends upwards in leaf epidermis in both treated and untreated plants (Fig. 5b, c). Under normal conditions, mesophyll peroxisomes are already less mobile than epidermal peroxisomes and other organelles in general (Figs 2 to 4, Figs S2 and S3). The mesophyll peroxisome velocities immediately following photorespiratory conditions are comparable to their speeds under normal growth conditions (Fig. 5c). Therefore, mesophyll peroxisomes may be “primed” to assume movement for their role in photorespiration, irrespective of photorespiratory status. In the dark, when minimal photosynthesis and photorespiration occur, peroxisomes are found in mitochondrion–chloroplast–peroxisome complexes in higher proportion than mitochondria (Oikawa et al. 2015), which corroborates our prediction.

Yet, despite increased interactions with chloroplasts (and mitochondria) during photorespiration (Jaipargas et al. 2016; Midorikawa et al. 2022), peroxisomes are faster, on average, in the mesophyll in treated samples, possibly attributable to later time points during recovery. Further, peroxisomes have a mildly higher average velocity after high-light treatment in nonphotosynthetic root tissue (Fig. 5b), consistent with previous findings that peroxisomes are more mobile in response to light (Oikawa et al. 2015). Thus, peroxisome motility may be more responsive to internal cues or stimuli independent of the chloroplasts, such as ROS accumulation observed in response to stress like cadmium treatment (Rodriguez-Serrano et al. 2009).

In summary, tracking thousands of organelles simultaneously allowed for direct spatial comparison across organelle identity and cell type and with acute temporal resolution for precise detection of subtle shifts in movement patterns. Using robust multifactorial quantification, we comprehensively characterized heterogeneous organelle motility and demonstrated that organelle motility is differentially and specifically modulated according to organelle identity, cell type, and physiological function. Our findings lead us to propose that organelle-specific and cell-dependent regulation of actomyosin-mediated movement is the predominant mode of organelle motility, which can subsequently modulate cytoplasmic streaming for efficient and responsive subcellular dynamics. The quantitative descriptions of the nuanced nature of plant organelle movement will enable future work to uncover molecular regulation of the dynamic organelle-specific motility.

## Materials and methods

### Plant materials and growth conditions

Tobacco (*N. tabacum*) and Arabidopsis (*A. thaliana*) Col-0 were used in this study. The *MyoXI-3KO* and three independent transgenic lines of WT expressing pLB187 (Huang et al. 2026) were generated by floral dipping (Zhang et al. 2006). Additional marker lines for mitochondria (mt-ck, CS16262) and peroxisomes (px-ck, CS16259) (Nelson et al. 2007) were obtained from the Arabidopsis Biological Resource Center (ABRC). Seeds were sterilized first with 95% ethanol for 5 min and then 30% bleach with 0.02% Triton X-100 for 20 min and washed 3 times with water. The seeds were then sown on 0.8% agar plates [½ Murashige and Skoog {MS} medium, 1% sucrose] with 25 mg/L hygromycin or kanamycin for selection, stratified in the dark at 4˚C for 3 d, and then moved to a 12 h light/dark photoperiod for 2 weeks.

### Transient expression in tobacco

*GmMan1* ^49aa^-eCFP (ABRC G-cb/CD3-962), *ScCOXIV*^29aa^-eYFP (ABRC mt-yk/CD-989), mScarlet-I-SRL, and Lifeact-eGFP (pLB187) were used as Golgi, mitochondrial, peroxisomal, and F-actin markers, respectively (Nelson et al. 2007; Koenig et al. 2023; Huang et al. 2026). Tobacco infiltration was performed as described previously (Jiang et al. 2025), with all markers combined in equal volumes.

### Photorespiratory conditions treatment

Two-week-old Arabidopsis with mitochondrial or peroxisomal markers were grown on ½ MS agar plates wrapped in micropore tape under 12/12 light/dark photoperiod and 100 µmol/m^2^s light. The photorespiration-treated plates were moved to a 700 µmol/m^2^s chamber after 2 weeks, while plates under normal conditions remained in the 100 µmol/m^2^s chamber for a third week. The micropore tape of the photorespiration-treated plates was replaced with 3 layers of parafilm to restrict gas exchange 48 h prior to imaging. Plates were removed from the growth chamber one at a time and transferred to the microscope in approximately 5 min. Each plate was rewrapped and placed in a 100 to 200 µmol/m^2^s growth chamber between samples. Samples were imaged in a randomized order, alternating between capturing each organelle and tissue type (Fig. S5). All samples were imaged within an hour after the photorespiration-inducing treatment.

### Microscopy

To compare organelle morphology and abundance, Golgi, peroxisomes, mitochondria, actin, and chloroplasts were visualized in the same tobacco cells on a Leica Stellaris 5 confocal laser scanning microscope. The excitation/emission wavelengths for each marker were as follows: Golgi (eCFP) 448 nm/455 to 485 nm, actin (eGFP) 489 nm/494 to 521 nm, mitochondria (eYFP) 514 nm/540 to 588 nm, peroxisomes (mScarlet-I) 587 nm/600 to 640 nm, and chloroplasts (chlorophyll autofluorescence) 448 nm/687 to 730 nm.

To evaluate organelle distribution in relation to F-actin, Golgi, peroxisomes, mitochondria, actin, and chloroplasts were visualized in the same tobacco cells on a Zeiss 980 with Airyscan 2 confocal laser scanning microscope. A lambda series and spectral unmixing were used to acquire the actin (eGFP) signal to minimize crossbleeding from the mitochondria (eYFP). Briefly, samples were excited at 488 nm, and images were captured at 12 emissions across 499 to 605 nm. Then, the same Z coordinates were used to capture Golgi (eCFP, 445 nm/458 to 480 nm), mitochondria (eYFP, 514 nm/517 to 561 nm), peroxisomes (mScarlet-I, 561 nm/577 to 603 nm), and chloroplasts (chlorophyll autofluorescence, 639 nm/653 to 668 nm) using standard confocal laser imaging.

To assess organelle motility, tobacco Golgi, mitochondria, and peroxisomes were imaged in the same cells with a 3i spinning disk confocal microscope configured on a Zeiss Axio Observer platform. First, peroxisomes (mScarlet-I, 561 nm/580.5 to 653.5 nm), mitochondria (eYFP, 515 nm/505.5 to 578.5 nm), and Golgi (eCFP, 445 nm/445.5 to 518.5 nm) were each captured every 250 ms for 1 min in an abaxial epidermal cell. Then the Z-position was lowered into the spongy mesophyll, and time interval images were acquired again for each organelle.

The epidermis and mesophyll cells near the petiole of Arabidopsis seedling leaves were used to visualize peroxisomes (mScarlet-I or eCFP) and mitochondria (eCFP) with the same excitations and emissions specified above for mScarlet-I and eCFP on the 3i spinning disk confocal microscope (Intelligent Imaging Innovations Ltd). Images were captured every 250 to 500 ms for 1 to 5 min.

### Organelle tracking and analysis

All organelles were tracked using the ImageJ plugin MTrackJ (Meijering et al. 2012). Organelles were tracked across all frames of the video; if the organelle was no longer visible in the field of view, the track was ended, and a new track was started if the organelle reappeared in later frames. The tracks data generated by MTrackJ were used to analyze mean velocity, length (Len), and angle change (Δα). The *x–y* coordinates from the points data were used to construct a polygon of the convex hull and its calculated area with R, adapted from published methods (Chustecki et al. 2021). To avoid bias for faster/slower populations of organelles, nearly all organelles in each video (∼25 to 250 organelles per video) were tracked and analyzed. Each density plot or violin plot represents 120 to 930 organelles from ≥3 biological replicates across cell-type and/or environmental condition, and each experiment was repeated at least twice.

## Supplementary Material

kiag119_Supplementary_Data

## Data Availability

Data available on request.
