# Supplementary material for: Multifactorial analysis of simultaneous organelle movement reveals cell-specific motility of peroxisomes and mitochondria
Source: Plant Physiol. 2026 Feb 28;200(3):kiag119. doi: 10.1093/plphys/kiag119 (PMC13012880; doi:10.1093/plphys/kiag119)
Supplement: kiag119_Supplementary_Data [file kiag119_supplementary_data.zip › Supplementary Figures.pdf]

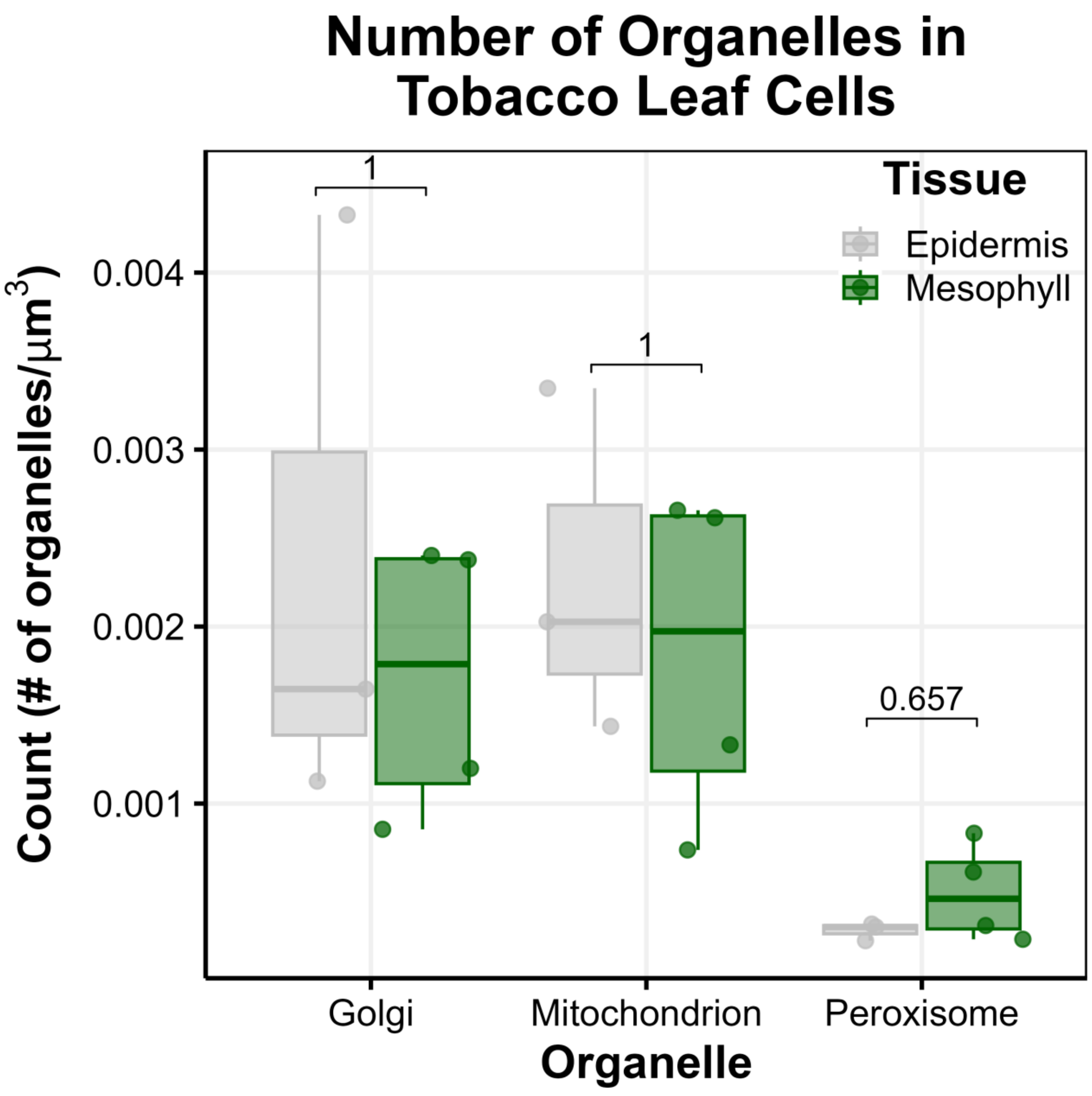

Supplemental Figure S1. The relative number of organelles is similar between cell types in tobacco leaves. Golgi, mitochondria, and peroxisomes were counted in Z stack images of the cortexes of tobacco leaf epidermis and mesophyll, and the total number of each was normalized to the volume of the image, represented by boxplots (center line, median; box limits, upper and lower quartiles; whiskers, 1.5x interquartile range; points, individual datapoints). Significance was determined by a Bonferroni-corrected Welch's t-test and adjusted p-values are reported on the graph.

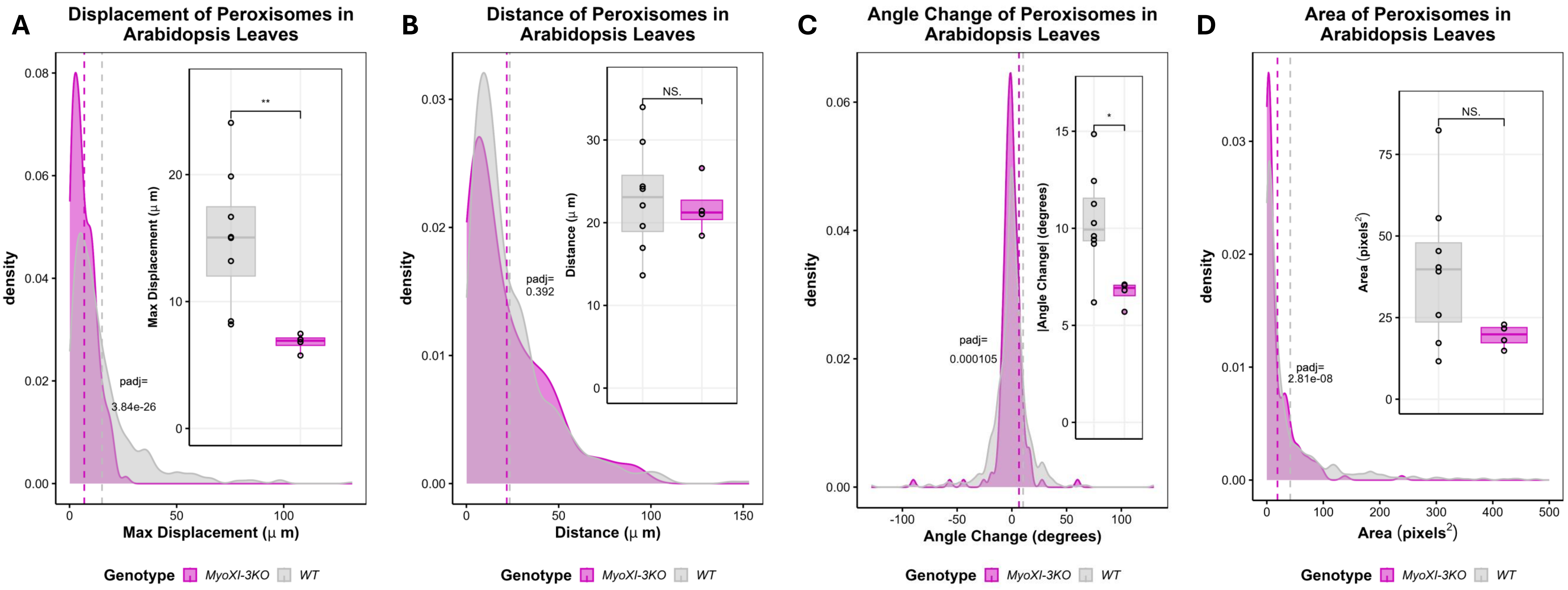

Supplemental Figure S2. Peroxisome movement is predominantly dependent on myosin motors. To determine whether the peroxisome motility factors measured are actomyosin dependent, peroxisomes in leaf epidermis of wildtype (WT, gray) and myosin triple knockout (*MyoXI-3KO*, pink) lines were visualized with an mScarlet-I-Ser-Arg-Leu and tracked. The displacement **(A)**, distance **(B)**, angle change **(C)**, and area **(D)** were calculated based on their coordinates from MTrackJ. The individual motility factors for each organelle track are shown as a density plot with the average for each organelle designated by a vertical dotted line, and the average motility across each plant sample is plotted as an inset boxplot (center line, median; box limits, upper and lower quartiles; whiskers, 1.5x interquartile range; colored points, outliers; black points, individual datapoints). Significance for density plots was determined by a Bonferroni-corrected Welch's t-test and adjusted p-values are reported on the graph. Significance for inset boxplots was determined by Student's t-test * $p<0.05$, ** $p<0.01$. Data represents ≥4 biological replicates, 660 and 170 peroxisomes in wildtype and *MyoXI-3KO,* respectively.

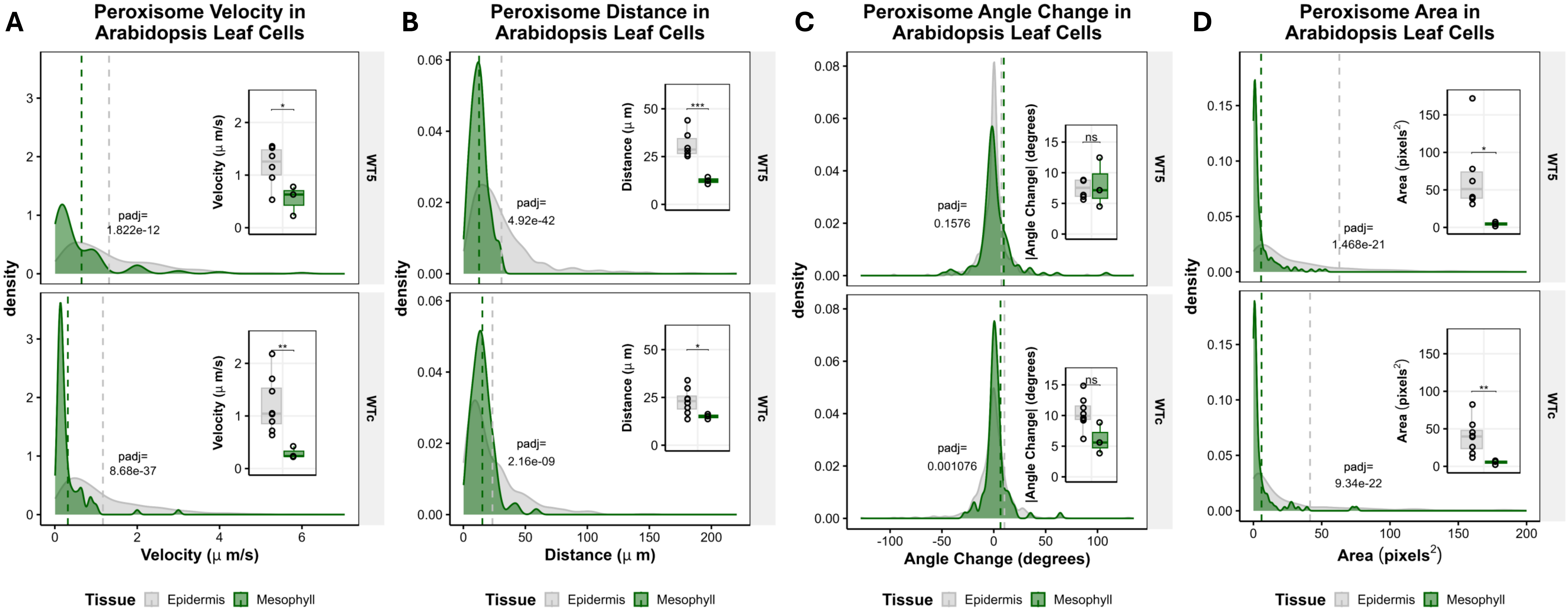

Supplementary Figure S3. Mesophyll peroxisomes are less mobile than epidermal peroxisomes in Arabidopsis leaves. The velocity **(A)**, distance **(B)**, angle change **(C)**, and area **(D)** of peroxisomes in Arabidopsis leaf epidermis is reported. The individual motility factors for each organelle track are shown as a density plot with the average for each organelle designated by a vertical dotted line, and the average motility across each plant sample is plotted as an inset boxplot (center line, median; box limits, upper and lower quartiles; whiskers, 1.5x interquartile range; colored points, outliers; black points, individual datapoints). Significance for density plots was determined by a Bonferroni-corrected Welch's t-test and adjusted p-values are reported on the graph. Significance for inset boxplots was determined by Welch's t-test * $p<0.05$, ** $p<0.005$, *** $p<0.0005$ Data represents 6-8 biological replicates and 630-676 organelles per independent transgenic line.

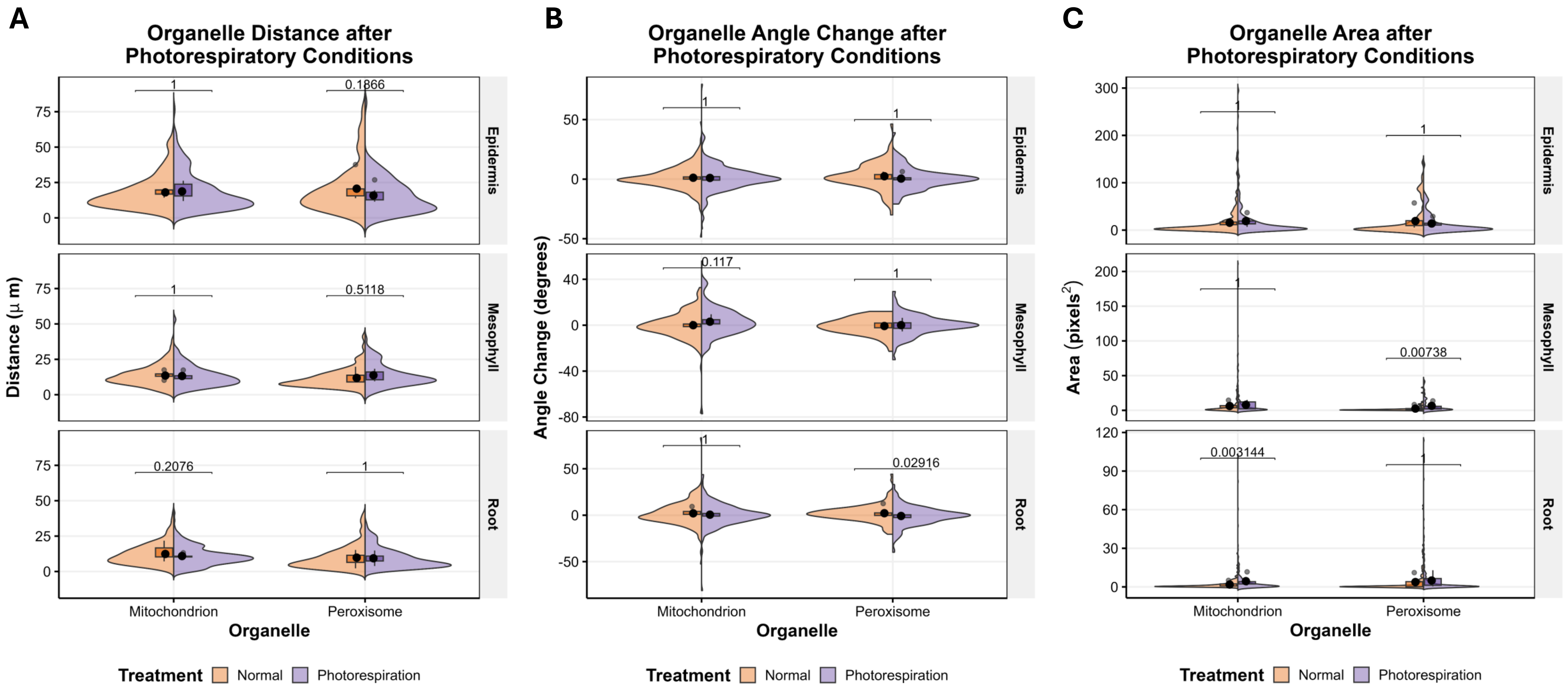

Supplementary Figure S4. Analysis of additional motility factors for the photorespiration experiment. The distance **(A)**, angle change **(B)**, and area **(C)** of peroxisomes (>700) and mitochondria (>1300) following normal and photorespiratory conditions were calculated based on their tracked coordinates. The motility factor for each organelle track is shown as a violin plot with the average for each organelle designated by a black dot with bars indicating the standard error, and the average across each plant sample (n=6) is shown as a boxplot (box limits, upper and lower quartiles; whiskers, 1.5x interquartile range; gray points, outliers). Significance was determined by a Bonferroni-corrected Welch's t-test and adjusted p-values are reported on the graph. Note, the appearance of negative motility measurements is a consequence of the untrimmed violin plot.

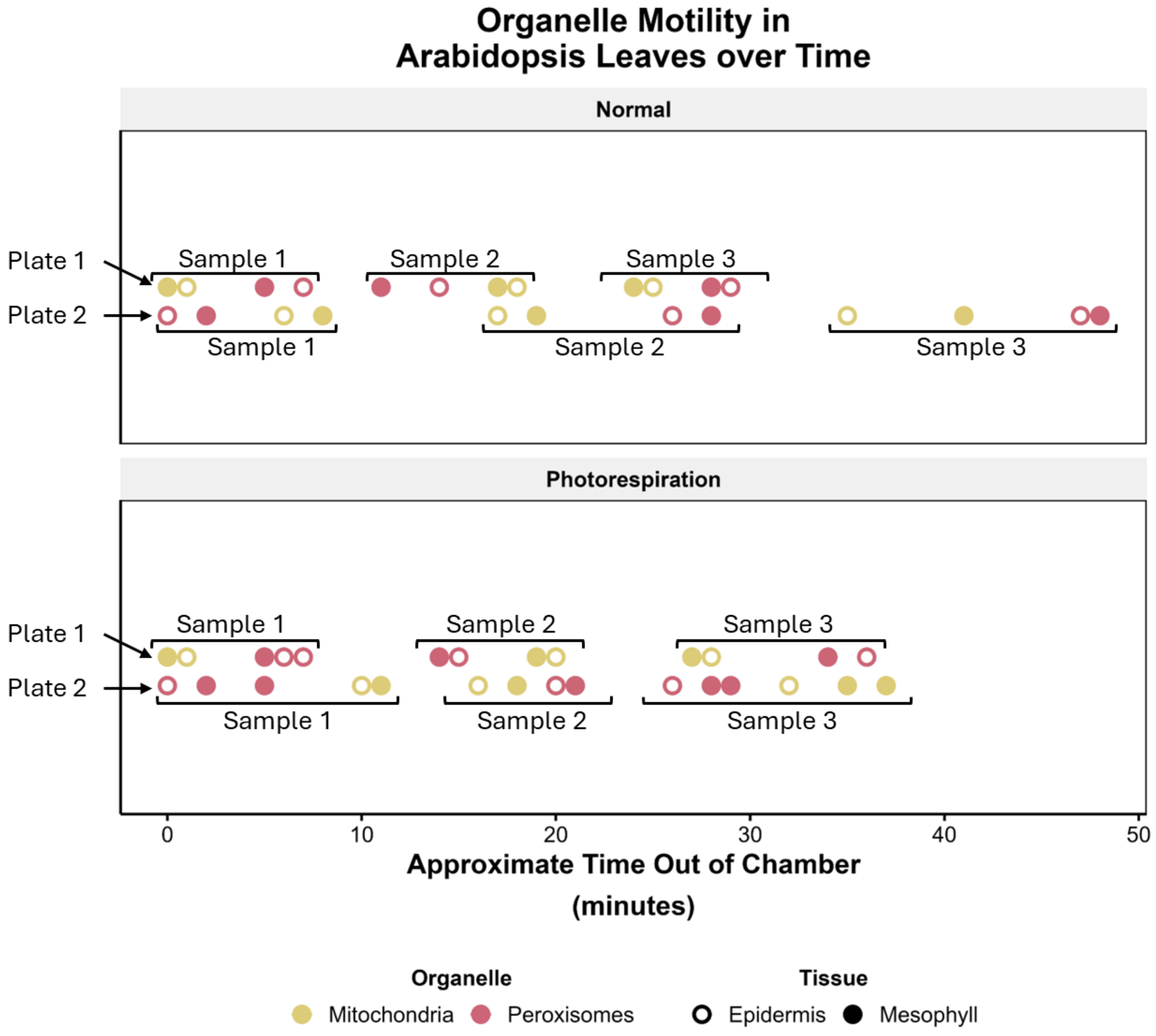

Supplementary Figure S5. Plot of image acquisition time for the photorespiration experiment. The image for the first sample of each plate (time=0) was captured 5-10 minutes after the plate was removed from the growth chamber. Image acquisition alternated randomly among the combinations of organelles (mitochondria, yellow; peroxisomes, red) and tissue types (epidermis, open; mesophyll, closed). Image acquisition for all samples from each plate were completed before retrieving the next plate from the growth chamber.
